# Relationship Between Physical Activity Level and Stress Perception: Exploring Factors During COVID-19 Pandemic

**DOI:** 10.34172/jrhs.2023.120

**Published:** 2023-09-29

**Authors:** Senay Çerezci-Duygu, Furkan Özdemir, Gökhan Karakaş

**Affiliations:** ^1^Department of Orthotics and Prosthetics, Gülhane Faculty of Health Sciences, University of Health Sciences, Ankara, Türkiye; ^2^Department of Biostatistics and Epidemiology, School of Rehabilitation, University of Social Welfare and Rehabilitation Science, Tehran, Iran; ^3^Faculty of Physical Therapy and Rehabilitation, Hacettepe University, Ankara, Türkiye

**Keywords:** Physical activity, Perceived stress, Pandemics, Barrier, Motivation, COVID-19

## Abstract

**Background:** After the difficulty of the pandemic process, managing the long-term effects that may occur after the coronavirus disease 2019 (COVID-19) is among the biggest concerns in the present era. This study aimed to explore factors affecting the physical activity level and investigate the relationship between physical activity level and stress perception of university students during the COVID-19 pandemic.

**Study Design:** A cross-sectional study.

**Methods:** Study data were collected via online survey, and volunteer participants completed the survey through the survey link between October 21 and December 31, 2021. Physical activity level was evaluated by "The International Physical Activity Questionnaire- Short Form" (IPAQ-SF), and stress level was evaluated by "Perceived Stress Scale" (PSS). The participants were asked questions about barriers and motivators for physical activity before and during the pandemic.

**Results:** The study included 444 participants (81.3% female and 18.7% male) with an average age of 21±2.95 years. The results showed a negative-significant linear relationship between perceived stress score and total physical activity, vigorous-intensity physical activity, and walking scores (r=-0.157, *P*<0.01; r=-0.16, *P*<0.01; r=-0.13, *P*<0.05 respectively). During the pandemic, the perception of insufficient finance became less important as a barrier (*P*=0.029), and healthcare professional (HCP) recommendation became more important as a motivator for physical activity than the pre-pandemic conditions (*P*=0.035).

**Conclusion:** The findings indicated that it is possible to reduce the level of perceived stress by increasing the level of physical activity. Current research will be a key for increasing and maintaining physical activity and reducing perceived stress.

## Background

 The coronavirus disease 2019 (COVID-19) pandemic was first detected from an outbreak in Wuhan, China, in December 2019. The virus spread worldwide in a short time, and then lockdowns and measures were taken to close all non-essential services in many countries. Applied pandemic measures helped to minimize contact, prevent virus transmission, and also had direct effects on health and well-being.^1^ Although the effects during the pandemic are challenging, managing the long-term effects that may occur after COVID-19 is among the major concerns today.^2^

 The major observable concern during the pandemic was a decreased amount of physical activity and increased sedentary behavior.^3^ In addition to the physical effects, psycho-social concerns were also discussed frequently during the pandemic process. Mental health disorders developed in people who had no psychiatric or psychological disorder in the past and progressed easily. A large part of the concerns focused on the young population with high psychological sensitivity.^4^ Physical activity is one of the basic and effective methods for the treatment of psychological disorders that are associated with stress. It is known that people with higher levels of physical activity tend to be less anxious and depressed.^5^

 A decrease in physical activity and an increase in psychological distress have been an observable fact in the COVID-19 pandemic.^6-8^ University students aged 18-29 are one of the groups that have become the focus of concerns with the sudden change in their active lifestyles.^9^ University students as young adults are expected to be particularly psychologically sensitive, and one of the keys to improving the health of this population is to encourage physical activity.^5^ Examining the factors related to physical activity will contribute to improving the long-term effects that can be observed after COVID-19.

 Therefore, the aim of this study was to examine the factors affecting the physical activity level of university students during the COVID-19 pandemic and investigate the relationship between physical activity level and stress perceptions. The results obtained from this research can reveal the relationship between physical activity and perceived stress levels and determine the change in barriers and motivators for physical activity during the pandemic. Identifying barriers and motivators for physical activity in the young population, including university students will be a key to promoting physical activity. Moreover, the results of the current research will be a key to increasing and maintaining well-being after the pandemic.

## Methods

###  Study Design

 This study was planned as cross-sectional. The study protocol was approved by Baskent University Institutional Review Board and Ethics Committee Board (Project no: KA21/378). To achieve a small margin of error based on a population size of university students with a population of approximately 7 million, a total of 444 participants were included in the study for a 5% margin of error with at least a 95% confidence interval.

 Data were collected over a monthly data collection period (October 21 to December 31, 2021). Consent was obtained by online survey, and volunteer participants completed the survey via the survey link shared on social media accounts. The study was conducted at universities in Turkey, and the online forms were prepared in Turkish. The online survey was open to all university students who agreed to participate voluntarily and whose native language was Turkish. Within the scope of the study design, surveys with appropriate validity and reliability in Turkey were preferred.

###  Inclusion Criteria

 Students registered at the university in the 2019-2020 Spring, 2020-2021 Fall, and 2020-2021 Spring semesters were included in the study, where online education was provided due to the COVID-19 pandemic. The inclusion criteria were being between the ages of 18-29, being university students, having an awareness of physical activity, and maintaining education through the online system during the pandemic process. The exclusion criteria were being in a department that did not switch to online education during the pandemic process or not being a university student within the specified time frame.

###  Outcome Measurements

 When participants opened the link to the online survey, there were questions regarding compliance with the inclusion criteria. If the participants met the inclusion criteria, they could continue with the main survey, which included demographic information and outcome measurements. The demographic characteristics questions were age, height, weight, gender, and whether they were educated in the city where their family lived. The outcome measurement questions after demographic characteristics are as follows:

###  Physical Activity

 The International Physical Activity Questionnaire-Short Form (IPAQ-SF) was used to quantify health-related physical activity. IPAQ-SF records the activity of four intensity levels: 1) vigorous-intensity activity such as aerobics, 2) moderate-intensity activity such as leisure cycling, 3) walking, and 4) sitting. Frequency (measured in days per week) and duration (time per day) are collected separately for each specific type of activity.

 Participants were asked the “last 7-day recall” version of the International Physical Activity Questionnaire Short Form (IPAQ-SF) for physical activity surveillance. Data collected with IPAQ was reported as a continuous measure and reported as median metabolic equivalent (MET) minutes. Median values can be computed for vigorous-intensity activities, moderate-intensity activities, and walking using the following formulas:

- Vigorous MET-minutes/week = 8.0 × vigorous-intensity activity ‘minutes’ × vigorous-intensity ‘days’
- Moderate MET-minutes/week = 4.0 × moderate-intensity activity ‘minutes’ × moderate intensity ‘days’
- Walking MET-minutes/week = 3.3 × walking ‘minutes’ × walking ‘days’
- A combined total physical activity MET-min/week was computed as the sum of vigorous + moderate + walking MET-min/week scores.^10,11^

###  Stress level

 Perceived Stress Scale (PSS) was used to quantify psychological stress levels. PSS items were designed to tap how unpredictable, uncontrollable, and overloaded participants find their lives. The scale also includes a number of direct queries about current levels of experienced stress. Participants evaluated each item on a 5-point Likert-type scale ranging from “never (0)” to “very often (4)” and were asked to take the last month as a reference for evaluation.^12,13^

###  Barriers and motivators to physical activity

 To identify barriers and motivators for physical activity, participants were asked to report barriers and motivations at the present time and 6 months prior (based on retrospective recall) to the COVID-19 pandemic using a multiple-choice list. The barriers and motivators evaluated in the current study were previously investigated and were found to significantly affect physical activity levels.^4,14^

###  Data analysis

 The analysis was completed by transferring the study data to the IBM SPSS Statistics 26 software. While evaluating the data, frequency distributions for categorical variables and descriptive statistics (Mean ± SD, median) for numerical variables were calculated. Then, the Kolmogorov-Smirnov normality test (n > 50) was applied to all measurements. According to the result of the test, IPAQ measurements did not provide the assumption of normality, while the perceived stress score provided the assumption of normality. For this reason, Spearman’s rho correlation coefficient, which is a nonparametric test, was used to determine the degree of non-causal relationships between two numerical variables. The relationship between two dependent categorical variables was examined using the McNemar Test. Multiple linear regression analysis was also applied using the Forward Method to determine whether the barrier and motivational items affected the IPAQ score during the COVID-19 pandemic. Furthermore, statistical significance was set at the 0.05 a-level.

## Results

###  Sample characteristics

 The study included 444 participants with an average age of 21 ± 2.95 years (min = 18, max = 28) and an average body mass index (BMI) of 21.93 ± 3.5 kg/m^2^ (min = 15.42, max = 39.79). There were 361 female (81.3%) and 83 male (18.7%) participants. Moreover, 189 (42.6%) of the participants were studying in the city where their families lived, while 255 (57.4%) were studying in a city far from their families.

###  Physical activity and perceived stress

 Descriptive statistics related to physical activity and perceived stress levels during COVID-19 were presented in Table 1. It was observed that the IPAQ measurements used as a measurement tool in the study were not normally distributed, and the PSS was normally distributed. Therefore, Spearman’s rho correlation coefficient was used to examine the relationship between scores (Table 2).

**Table 1:** Descriptive statistics of physical activity and perceived stress levels during COVID-19 pandemic based on IPAQ

**IPAQ**	**Mean**	**SD**	**Median**	**Min**	**Max**
Vigorous MET-min/week	2197.24	14023.97	0	0	288000
Moderate MET-min/week	744.13	1674.07	2.50	0	13440
Walking MET-min/week	1330.63	2489.45	693	0	38280
IPAQ total MET-min/week	4278.11	16341.501	1628	0	327960
IPAQ sitting Time (min/week)	365.03	226.21	300	20	1200
Perceived stress scale	28.62	7.10	28	9	51

*Note*. COVID-19: Coronavirus disease 2019; IPAQ: International physical activity questionnaire; SD: Standard deviation; MET: Metabolic equivalent.

**Table 2:** Examining the relationships between physical activity level and perceived stress scale during COVID-19 pandemic based on IPAQ

**Variables**	**1**	**2**	**3**	**4**	**5**
1. IPAQ vigorous MET-min/week	1.000				
2. IPAQ moderate MET-min/week	0.375	1.000			
3. IPAQ walking MET-min/week	0.430	0.21	1.000		
4. IPAQ total MET-min/week	0.768	0.564	0.831	1.000	
5. Perceived stress scale score	-0.160	-0.039	-0.130	-0.157	1.000

*Note*. COVID-19: Coronavirus disease 2019; IPAQ: International physical activity questionnaire; MET: Metabolic equivalent.

###  Barriers and motivators for physical activity

**Figure 1:**
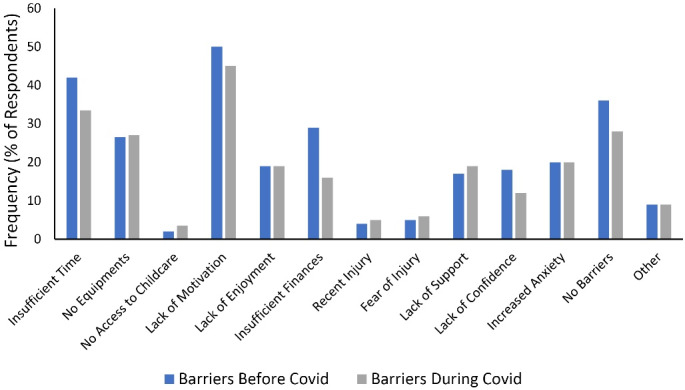
Distribution of “yes” responses to barriers to exercise items before and during COVID-19 pandemic

**Figure 2:**
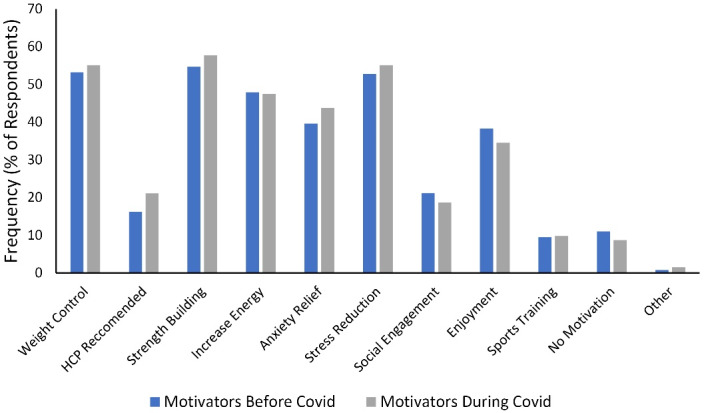
Distribution of “yes” responses to motivators to exercise items before and during COVID-19 pandemic

 The current study examined whether there is a difference between the answers to the barrier and motivation items for exercise before and during the COVID-19 pandemic. Table 3 and Figure 1 present barrier items, and Table 4 and Figure 2 depict motivation items. According to the result of the McNemar test, a statistically significant difference was observed between the yes answer given by the participants to the barrier item “insufficient finances” and the motivation item “healthcare professional (HCP) recommended” before and during the COVID-19 pandemic.

**Table 3:** Examining the Relationships Between Self-reported Barriers to Exercise Before and During COVID-19 Pandemic(N = 200)

**Variables**	**Barriers before ** **COVID-19 pandemic**		**Barriers during** **COVID-19 pandemic**		***P***** value**
	**Number**	**Percent**	**Number**	**Percent**	
Insufficient time	84	42.0	67	33.5	0.086
No equipment	53	26.5	54	27.0	1.000
No access to childcare	4	2.0	7	3.5	0.549
Lack of motivation	100	50.0	90	45.0	0.382
Lack of enjoyment	19	9.5	19	9.5	1.000
Insufficient finances	29	14.5	16	8.0	0.029
Recent injury	4	2.0	5	2.5	1.000
Fear of injury	5	2.5	6	3.0	1.000
Lack of support	17	8.5	19	9.5	0.868
Lack of confidence	18	9.0	12	6.0	0.345
Increased anxiety	20	10.0	20	10.0	1.000
No barriers	36	18.0	28	14.0	0.322
Other	9	4.5	9	4.5	1.000

*Note*. COVID-19: Coronavirus disease 2019; IPAQ: International physical activity questionnaire; MET: Metabolic equivalent.

**Table 4:** Examining the relationships between self-reported motivators to exercise before and during COVID-19 pandemic (n = 265)

**Variables**	**Barriers before ** **COVID-19 pandemic**		**Barriers during** **COVID-19 pandemic**		***P *****value**
	**Number**	**Percent**	**Number**	**Percent**	
Weight control	141	53.2	146	55.1	0.576
Recommendation of health care professional	43	16.2	56	21.1	0.035
Strength building	145	54.7	153	57.7	0.382
Energy increase	127	47.9	126	47.5	1.000
Anxiety relief	105	39.6	116	43.8	0.161
Stress reduction	140	52.8	146	55.1	0.512
Social engagement	56	21.2	49	18.6	0.392
Enjoyment	101	38.3	91	34.5	0.275
Sports training	25	9.5	26	9.8	1.000
No motivation	29	11.0	23	8.7	0.307
Other	2	0.8	4	1.5	0.624

 A multiple linear regression model was established to examine whether the barrier items affect the IPAQ score. The model has been tested with the Forward Method. The forward method starts with the structure where no variables are in the model. It adds the variable with the highest partial correlation with the dependent variable to the model, and the addition process to the model ends when it does not meet the specified criterion. The established regression model was confirmed with only one of the barrier items. The regression model in which the “insufficient time” expression is an independent variable and the IPAQ score is a dependent variable is statistically significant (F = 5.324, *P* = 0.022). Moreover, 1.5% of the change in the IPAQ score is explained by the independent variable in the model (Adjusted R^2^ = 0.015). Accordingly, the IPAQ score of the students who answered yes to the statement was 5 726 896 (B) times higher than that of the students who said no. Furthermore, the effect level of the expression on the IPAQ score was 0.135 (Std. Beta).

 A multiple linear regression model was established to examine whether the motivation items affect the IPAQ score. The model has been tested with the Forward Method. The established regression model was confirmed with only one of the motivation items. The regression model in which the “HCP recommended” expression is an independent variable and the IPAQ score is a dependent variable is statistically significant (F = 4.737, *P* = 0.03). In addition, 1.3% of the change in the IPAQ score is explained by the independent variable in the model (Adjusted R^2^ = 0.013). Accordingly, the IPAQ score of the students who answered yes to the statement was 6596,049 (B) times higher than that of the students who said no. Furthermore, the effect level of the expression on the IPAQ score was 0.130 (Std. Beta).

## Discussion

 It is known that this is neither the first nor the last pandemic. It is evident that problems caused by physical inactivity are critical, and young adults are the group most affected by complications caused by the COVID-19 pandemic.^15,16^ The present study investigated the relationship between physical activity and stress perception of young adults studying at university during the pandemic period and also examined barriers and motivators that had the potential to affect physical activity levels.

 Mental wellness was one of the main concerns during the COVID-19 pandemic.^17^ It was stated that the pandemic is a serious contributor to anxiety and stress in the young population, and physical activity was the most documented preventative factor.^17-20^ Study results indicated that during the pandemic, the perceived stress level decreased with vigorous physical activity, walking, or total physical activity level. Considering university students, increasing the level of physical activity for stress management will be an effective and accessible method without any adverse effects. The increase in the mentioned physical activity level can be achieved at a certain level even by increasing the daily amount of walking.

 There is limited source about the effects of the COVID-19 pandemic on barriers and motivators for physical activity, which has been shown to reduce perceived stress levels. In a self-reported study that involved individuals from all age groups, weight control and stress reduction were the motivation factors directing individuals to activity.^4^ Our study results support previous study findings. In the current study, among the motivation factors for physical activity before and after the pandemic, the first three based on the number of people were strength building, weight control, and stress reduction. It was observed that this proportional superiority does not change with the pandemic process, and the three reported motivation items are still valid during COVID-19. Although weight control and stress reduction are the most common reasons for engaging in physical activity, it is important to be aware that the perception of stress management is the highest. The current study also proved that increasing the level of physical activity would be effective for stress management.

 HCP is considered reliable for making recommendations for physical activity and has a key role in promoting physical activity, especially among populations at high risk for physical inactivity.^21^ HCP recommendation is an important component for increasing the level of physical activity in various diseases and different age groups, but generally, it has not been sufficiently addressed. In this view, the HCP recommendation, which was also important before the pandemic, has become a more important motivator for increasing the level of physical activity during and possibly after the pandemic process.

 It was found that the physical activity levels of university students decreased during the COVID-19 pandemic.^22^ In previous studies, it was reported that taking person-specific physical activity recommendations increases physical activity levels.^23^ Similarly, in the multiple linear regression model, it was seen that the effect of the HCP recommendation statement on the level of physical activity is statistically significant, and the physical activity score of the students who answered yes to the statement is higher than that of the students who said no. This indicates that determining a goal for reaching the physical activity level determined by a HCP may be an effective method for increasing the physical activity level of college students.

 Our results and the results of previous studies suggest that physical activity counseling by HCP is important, and the benefit from physical activity counseling increases significantly with the pandemic process. It can be predicted that the use of physical activity counseling by HCP in the young population studying at university whose inactivity rate has increased during the pandemic process can also prevent long-term negative effects.

 In a previous study, it was reported that the lack of motivation and inappropriate equipment, space, and facilities are the most common factors for physical activity barriers during the COVID-19 pandemic.^24^ In our study, we found similar results regarding the lack of motivation and equipment, but conversely, we found that insufficient time is among the first three barrier expressions with the highest proportional distribution both before and during the pandemic. Although it was expected that people would be more advantageous in terms of time during the pandemic restrictions, the fact that the study was conducted on university students and perhaps the lack of time was a learned barrier continued the perception that there was not enough time for physical activity during the pandemic.

 When the physical activity barriers were examined, the only meaningful change with the pandemic process was in the insufficient finances. The mentioned significant change showed that the number of people who saw insufficient financial resources as a barrier to physical activity decreased during the pandemic process. We think that one of the reasons for this situation may be that 57% of the participants lived in different cities and returned to the cities where they lived with their families as distance education started. Although financial status was not questioned in detail within the scope of the current study, it is a predictable result that the financial difficulties arising from accommodation may have decreased for university students during the distance education in which the study was conducted. Given the described impact of insufficient finances, increasing physical activity areas for students after the pandemic and positive discrimination in favor of the young population in a way that puts less financial burden on access to these areas can reduce the perception of insufficient financial resources as a barrier in the long term. In addition, in the regression model examining the relationship between physical activity and barrier items, it was observed that the effect of insufficient time on the level of physical activity is statistically significant, and the physical activity level of the students who said yes to the statement is higher than that of the students who said no (Std. Beta: 0.135). It can be stated that the participants with a higher level of physical activity have time awareness and expect more in terms of time.

 Insufficient time barrier and insufficient time perception have been the issues discussed before the pandemic, and different suggestions and methods have been proposed to avoid possible negative influences on physical activity participation.^25,26^ Among the suggestions mentioned, we assume that the items that may be effective for university students are increasing the accessibility of sports fields in the education area for students, adding physical activity to daily routine, and identifying available time slots in daily routine for physical activity.

 In addition to our conclusions from the current study, we also encountered some limitations. First of all, barriers and motivators for physical activity were asked before and during the pandemic. Since the study was conducted during the pandemic, the barriers and motivators during the pandemic were evaluated by questioning the current situation, but before the pandemic, they were based on retrospective recall. Although there are debates about the retrospective recall method, it was used in the current study for the cross-sectional design. Secondly, the definition of “HCP recommendation” which is one of the motivators is not clear to the respondent: did the recommendation come from the government’s health department or did students get the advice from social media, mainstream media, or alternative media, what were the characteristics and education of the people they defined as HCP, or were the recommendations personalized. This can be defined in more detail in subsequent studies. Finally, a self-reported web-based survey was used to collect data; therefore, the accuracy of the responses could not be verified. In addition, the large confidence interval in our sample calculation will help minimize the impact of individual bias in reporting.

**Highlights**

- The level of perceived stress during the COVID-19 pandemic can be reduced by improving vigorous physical activity, walking, or total physical activity levels.
- During the COVID-19 pandemic, the “Health Care Professional Recommendation” became more important as a motivator for physical activity than the pre-pandemic conditions.
- It is known that this is neither the first nor the last pandemic. Therefore, current research will be a key to increasing and maintaining physical activity and reducing perceived stress in the future.

## Conclusion

 In conclusion, our findings point out that it is possible to reduce the level of perceived stress by increasing the level of physical activity. The importance of increasing accessibility and consultation to HCP to increase physical activity motivation increased during the pandemic lockdowns and physical distancing measures. In addition, our study revealed the most perceived motivations and barriers to physical activity during a global pandemic. These findings have been definitive for both relevant populations and healthcare professionals. These findings will be a key for increasing and maintaining physical activity levels and reducing perceived stress after the pandemic and lockdowns that may occur in the future.

## Acknowledgments

 None.

## Authors’ Contribution

**Conceptualization:** Senay Çerezci-Duygu, Furkan Özdemir.

**Data curation:** Furkan Özdemir.

**Formal analysis:** Senay Çerezci-Duygu, Furkan Özdemir.

**Funding acquisition:** Senay Çerezci-Duygu.

**Investigation:** Furkan Özdemir, Gökhan Karakaş.

**Methodology:** Senay Çerezci-Duygu, Furkan Özdemir.

**Project administration: **Senay Çerezci-Duygu, Furkan Özdemir.

**Resources:** Senay Çerezci-Duygu.

**Software:** Furkan Özdemir, Gökhan Karakaş.

**Supervision:** Senay Çerezci-Duygu.

**Validation:** Senay Çerezci-Duygu, Furkan Özdemir.

**Visualization:** Furkan Özdemir, Gökhan Karakaş.

**Writing–original draft:** Furkan Özdemir, Gökhan Karakaş.

**Writing–review & editing:** Senay Çerezci-Duygu.

## Competing Interests

 The authors declare no conflict of interests.

## Ethical Approval

 The study’s protocol was approved by the Baskent University Institutional Review Board and the Ethics Committee Board (Project no: KA21/378). In accordance with the Helsinki Declaration, written informed consent was obtained from each student before inclusion.

## Funding

 This research received no external funding.
